# Four‐dimensional tissue deformation reconstruction (4D TDR) validation using a real tissue phantom

**DOI:** 10.1120/jacmp.v14i1.4012

**Published:** 2013-01-07

**Authors:** Martin Szegedi, Jacob Hinkle, Prema Rassiah, Vikren Sarkar, Brian Wang, Sarang Joshi, Bill Salter

**Affiliations:** ^1^ Department of Radiation Oncology University of Utah Salt Lake City UT USA; ^2^ Scientific Computing and Imaging (SCI) University of Utah Salt Lake City UT USA

**Keywords:** validation, real tissue phantom, tissue deformation, SBRT, 4D CT, 4D dose

## Abstract

Calculation of four‐dimensional (4D) dose distributions requires the remapping of dose calculated on each available binned phase of the 4D CT onto a reference phase for summation. Deformable image registration (DIR) is usually used for this task, but unfortunately almost always considers only endpoints rather than the whole motion path. A new algorithm, 4D tissue deformation reconstruction (4D TDR), that uses either CT projection data or all available 4D CT images to reconstruct 4D motion data, was developed. The purpose of this work is to verify the accuracy of the fit of this new algorithm using a realistic tissue phantom. A previously described fresh tissue phantom with implanted electromagnetic tracking (EMT) fiducials was used for this experiment. The phantom was animated using a sinusoidal and a real patient‐breathing signal. Four‐dimensional computer tomography (4D CT) and EMT tracking were performed. Deformation reconstruction was conducted using the 4D TDR and a modified 4D TDR which takes real tissue hysteresis (4D ${\text{TDR}}_{\text{Hysteresis}}$) into account. Deformation estimation results were compared to the EMT and 4D CT coordinate measurements. To eliminate the possibility of the high contrast markers driving the 4D TDR, a comparison was made using the original 4D CT data and data in which the fiducials were electronically masked. For the sinusoidal animation, the average deviation of the 4D TDR compared to the manually determined coordinates from 4D CT data was 1.9 mm, albeit with as large as 4.5 mm deviation. The 4D TDR calculation traces matched 95% of the EMT trace within 2.8 mm. The motion hysteresis generated by real tissue is not properly projected other than at endpoints of motion. Sinusoidal animation resulted in 95% of EMT measured locations to be within less than 1.2 mm of the measured 4D CT motion path, enabling accurate motion characterization of the tissue hysteresis. The 4D ${\text{TDR}}_{\text{Hysteresis}}$ calculation traces accounted well for the hysteresis and matched 95% of the EMT trace within 1.6 mm. An irregular (in amplitude and frequency) recorded patient trace applied to the same tissue resulted in 95% of the EMT trace points within less than 4.5 mm when compared to both the 4D CT and 4D ${\text{TDR}}_{\text{Hysteresis}}$ motion paths. The average deviation of 4D ${\text{TDR}}_{\text{Hysteresis}}$ compared to 4D CT datasets was 0.9 mm under regular sinusoidal and 1.0 mm under irregular patient trace animation. The EMT trace data fit to the 4D ${\text{TDR}}_{\text{Hysteresis}}$ was within 1.6 mm for sinusoidal and 4.5 mm for patient trace animation. While various algorithms have been validated for end‐to‐end accuracy, one can only be fully confident in the performance of a predictive algorithm if one looks at data along the full motion path. The 4D TDR, calculating the whole motion path rather than only phase‐ or endpoints, allows us to fully characterize the accuracy of a predictive algorithm, minimizing assumptions. This algorithm went one step further by allowing for the inclusion of tissue hysteresis effects, a real‐world effect that is neglected when endpoint‐only validation is performed. Our results show that the 4D ${\text{TDR}}_{\text{Hysteresis}}$ correctly models the deformation at the endpoints and any intermediate points along the motion path.

PACS numbers: 87.55.km, 87.55.Qr, 87.57.nf, 87.85.Tu

## I. INTRODUCTION

Due to the increasing use of highly conformal radiation therapy treatments in the body (e.g., SBRT), motion management continues to grow in importance in radiation oncology. A widely used tool for characterizing patient‐specific organ/tumor motion is four‐dimensional computed tomography (4D CT). In addition to the accurate knowledge of patient‐specific organ motion gained by use of 4D CT imaging, another application of recent interest is the calculation of a so‐called ‘4D dose distribution’. A 4D dose distribution can be generated by calculating dose on the individual phases of the 4D scan and then remapping dose from all phases onto a common ‘reference’ phase, and finally summing for a total dose.

Since each binned CT phase of the 4D CT represents the patient slightly deformed from any other CT phase, deformable image registration (DIR) algorithms are essential to facilitating the remapping of dose from multiple CT phases onto a single reference phase for summation to generate the 4D dose distribution. B‐spline registration has been widely used for DIR in the context of radiation therapy.^(^ ^1^ ^)^ It generally involves interpolation/smoothing, which leads to reduced accuracy for large deformations, rendering the calculated deformation noninvertible and requiring a greater number of sampled anatomical points to maintain accuracy. Thus, the further away from the reference phase such algorithms operate, the greater the registration error would be — thus penalizing large deformations. More recently, DIRs have been proposed which employ a diffeomorphic motion model using splines,^(^ ^2^ ^)^ an approach which still penalizes larger deformations, but uses a chain of velocity fields from CT‐phase to CT‐phase to approximate a fluid flow model. Other authors have applied incompressible fluid flow models directly to phase‐binned 4D CT image data in order to find correspondences and to estimate voxel trajectories.^(^ ^3^ ^–^ ^5^ ^)^

Hinkle et al.^(^ ^6^ ^)^ extended such an algorithm in order to apply it to either raw CT projection data (if available) or CT images to estimate motion during the image reconstruction process in a maximum a posteriori (MAP)‐deformation algorithm, allowing for tracking of organ motion. We will refer to this practice of fitting a spatiotemporal motion model directly to raw imaging data as a 4D tissue deformation reconstruction (4D TDR). In order to alleviate DIR limitations, the 4D TDR performs a joint registration wherein a spatiotemporal motion model and a single image are matched simultaneously to all the other images. Regularization of the motion model may then be used to diminish the impact of image artifacts on the resulting motion estimate. The 4D TDR does not require the input to be full volumes, but can accept as input the raw time‐stamped projection data directly from the scanner.^(^ ^6^ ^)^ Due to the use of projections, a deformation reconstruction algorithm (and thus the 4D TDR) can “reconstruct” (using projection data) the images needed to accurately display deformation at any time t. If projection data is not available the use of already reconstructed images forces the algorithm to assume slow deformation (i.e., a static anatomy during the longer time needed to generate all projections of an image), and thus the 4D TDR can only assign a specific image to a specific time (ideally the time stamp of the image is right at the same time where half the time has passed to acquire all projections for this specific image). We would like to emphasize that the 4D TDR is not a deformation image registration (DIR) algorithm, which is limited by the predetermined number of phases (and thus images) and by the inherent potential for misaligned phase for some images. Such an algorithm can produce not only the best‐fit motion estimate, but also an anatomical volume image which is reconstructed during the motion estimation process. Artifacts resulting from binning errors are avoided in this model, since the raw images are never binned. This approach allows reconstruction of a motion path at any time or position while providing a lower signal to noise ratio.

Typical validations of motion models previously described employ one of two broad computational categories: mathematically‐based^(^ ^5^ ^,^ ^7^ ^,^ ^8^ ^)^ and/or proof of performance by comparison with manually delineated organ contours/landmarks derived from retrospective patient datasets. All of these authors, therefore, mention the difficulties in achieving accuracy for validation beyond interobserver uncertainties and, thus far, none has described voxel to voxel accuracy validation. The accuracy of deformation estimates is, therefore, generally expressed through the deviation measured for the two end state of motion when compared to a calculated estimation. All studies thus far assume perfect fit of the model to reality at the deformations start phase.

Brock^(^ ^9^ ^)^ had multiple institutions analyze a contrast‐injected liver study using 25 natural landmarks (vessel bifurcations) in the liver. Brock's work represents an extensive sample of tested DIR algorithms, with the best one resulting in an average 3D error of 1.8 mm between the start phase and the required end phase, called “vector magnitude”. In this case, the average error expresses the match of multiple landmarks at the CT‐phase endpoint. Deviations between DIR calculation and intermittent CT‐phase measurements on the motion path (i.e., before the motion path is completed) are usually neglected. In terms of the 4D dose calculation, an understanding of these errors is equally important. Even if multiple CT phases are analyzed, the calculation process involves the independent computation of multiple volume‐to‐volume registrations (i.e., the point trajectory model is only piecewise smooth and CT‐phase artifacts in any one image can drastically affect estimated point trajectories, falsifying the motion path). DIR, itself, further limits the capability of measuring deviations caused by a difference of the time–location relation, as estimated and measured. Thus, current studies are limited in their verification of measured landmark locations regarding their temporal–spatial accuracy, and therefore published errors often use deformation endpoints. Regardless of the method employed, it is obvious that, for the subsequently calculated 4D dose distribution to be accurate, the deformation calculated must be accurate on a voxel‐to‐voxel scale over the whole motion path and at any moment in time.

Previous validation studies, including that of Brock,^(^ ^9^ ^)^ evaluate the performance of DIR algorithms by comparing the position of high‐contrast feature points against “ground truth” positions. Such evaluations are useful in determining algorithms which fail to map easily identified points reliably, but offer little information about the performance of an algorithm in low‐contrast regions, such as the interior of the liver. Since DIR and 4D TDR algorithms each use image contrast to guide motion estimation, it is considerably more challenging to evaluate performance at low‐contrast points. In this work, we validate the performance of a 4D TDR algorithm in such a way. Even though it is infeasible to track as many low‐contrast points using fiducials as are commonly used in extracted‐feature studies, the additional challenge provided by the lack of contrast makes such validation quite useful.

We believe it reasonable to submit that the most meaningful validation of voxel‐to‐voxel accuracy for a given DIR or 4D TDR will require use of a deformable phantom capable of repeatable, realistic simulation of all important tissue specific behaviors, including voxel deformation and hysteresis. Further, not only is an end‐to‐end validation of motion needed, but so is a full analysis of the error in time and location of voxels. For this reason we have designed, constructed, and previously reported on a clinically realistic porcine liver phantom which is capable of producing patient‐equivalent tissue deformation.^(^ ^10^ ^)^ Here we use this real‐tissue 4D phantom to produce periodic 3D‐motion, equivalent to that typically observed in patients, to validate the free‐form diffeomorphism/smooth velocity flow 4D TDR model.^(^ ^6^ ^)^ By performing accurate measurements of the deformation in the phantom using 4D CT and electromagnetic tracking data, and comparing them against the deformation predictions, we directly evaluate the performance of this 4D TDR. We believe this to be the first time that such a time‐relevant voxel‐to‐voxel validation of deformation has been performed.

## II. MATERIALS AND METHODS

A previously described motion phantom containing a porcine liver lobe^(^ ^10^ ^)^ with fiducials embedded for voxel‐to‐voxel accuracy testing of our previously reported 4D TDR^(^ ^6^ ^)^ allows for a quantitative validation approach without interobserver influence. The phantom reproduces liver fiducial motion, which is equivalent to respiratory‐driven human liver fiducial motion measured in patients.^(^ ^11^ ^)^

### A. Data acquisition

**Figure 1:**
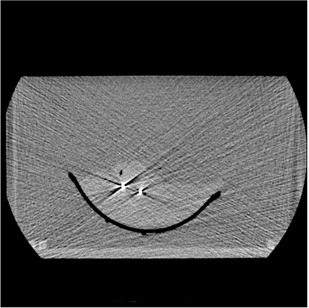
Typical axial slice of the prepared liver tissue phantom with two EMT transponders visible.

The phantom was prepared with a freshly explanted porcine liver containing three electromagnetic tracking (EMT) transponders (~ 8 mm long and 2 mm in diameter). A phantom motion controller that runs sinusoidal and irregular patient‐recorded breathing pattern, via a piston, was applied onto the liver. For this study, we independently used either a sinusoidal trace with a 6 s period or a patient trace, which was selected for its moderate irregularity in period and amplitude. The fiducials within the tissue define locations at which voxel to voxel tracking accuracy is directly validated. A surrogate marker box was placed on the chest‐motion platform of the phantom during 4D CT acquisition. A GE LightSpeed RT16 CT scanner (GE Healthcare, Waukesha, WI) with the real‐time position management (RPM) system (Varian RPM, Varian Medical Systems Inc., Palo Alto, CA)^(^ ^12^ ^–^ ^14^ ^)^ was used for the 4D CT acquisition. A description of the GE phase‐binned 4D CT acquisition and processing has been previously published by Pan et al.^(^ ^15^ ^)^ Images were acquired using 1.25 mm slices while maximizing the number of images that can be obtained for a 4D CT dataset. The GE system in our institution allows for a maximum of 3000 images within one 4D CT study. Due to technical settings, the image number of 4D CT ranged from 2880 to 2944 images for the acquisitions. A sample image, showing an axial image of the liver lobe with two EMT transponders can be seen in Fig. 1.

Image data were processed using the GE AW SIM MD software (version 7.6) (GE Healthcare) to generate 20 separate phase‐binned CT image sets, each with approximately 128–144 images, depending on the length of couch movement during data acquisition. This 4D CT data were then used to generate a complete motion path for each fiducial.

In order to confirm the accurate 4D CT measurement of fiducial motion and to understand the natural variation of motion within tissue, the phantom's complete motion was tracked for multiple periods using an EMT localizing and tracking system (Calypso Medical Technologies Inc., Seattle, WA), located in a separate treatment vault. In each case, the animation lasted approximately 10 minutes. The EMT‐generated data (10 Hz for individual transponders), utilizing a higher temporal positional acquisition rate than 4D CT, were compared to the 4D CT measured fiducial coordinates motion pattern and, ultimately, to the 4D TDR predicted motion pattern. Table 1 details the sequence of phantom image acquisitions using one liver.

**Table 1:** Sequence of imaging and tracking acquisition for one porcine liver.

*Acquisition Sequence*	*Animation Trace*
4D CT	Sinusoidal
4D CT	Patient
Calypso	Sinusoidal
Calypso	Patient

### B. Data processing

**Figure 2:**
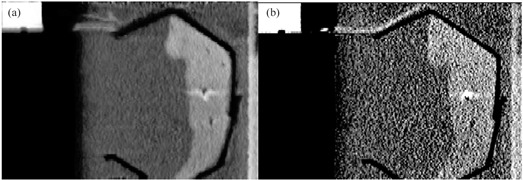
4D TDR reconstructed image (a) and according raw 4D CT image (b).

Data points in the phase sampled CT data were analyzed by one manual observer. Data points in the 4D TDR were acquired through software means. Two stationary reference point coordinates were used as basis for evaluation of measurement accuracy and as origins from which to measure all distances within and between 4D CT datasets, allowing comparisons from different CT phases. To create a spatiotemporal link between 4D CT and 4D TDR‐generated data, the piston position acts as a common reference point to map the 4D CT phase‐based data into the temporal/amplitude‐based frame of the 4D TDR. The EMT fiducials' locations were compared against the motion pattern generated by the respective fiducial in the phase binned 4D CT data. For visualization, Fig. 2(a) shows a sagittal 4D TDR reconstructed image containing the liver and animating piston compared to the raw sagittal 4D CT image in Fig. 2(b).

The 4D TDR‐predicted fiducial motion patterns were calculated, allowing for comparison with the 4D CT‐phase binned motion pattern. To confirm the accuracy of the 4D CT measurement, which represents the ‘ground truth’ for comparison with the 4D TDR, the shortest 3D distance of each EMT trace point to the interpolated CT‐phase binned motion track was calculated. Good agreement of the 4D CT with the high temporal resolution EMT indicates accurate (i.e., artifact free) 4D CT measurement of fiducial motion patterns.

#### B.1 Maximum a posteriori (MAP) 4D tissue deformation reconstruction (4D TDR)

**Figure 3:**
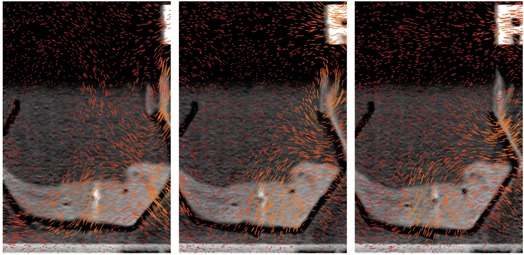
Three velocity fields of the tissue phantom undergoing a forward motion (images to be viewed from left to right).

We used all raw, time‐stamped 4D images to reconstruct and estimate deformations in anatomy. In order to estimate a 4D image, we employed the method previously outlined in detail by Hinkle et al.^(^ ^6^ ^)^ Using the breathing trace from the RPM system, along with the data time stamps, the raw image data were tagged with a breathing‐signal amplitude. The algorithm employs a steepest descent scheme, which maximizes the posterior likelihood under a prior distribution placed on the velocity fields. Our aim is to receive smooth velocity fields and a realistic and feasible deformation solution. An example of such velocity fields can be seen in Fig. 3.

For any given RPM breathing signal amplitude, the velocity fields are then used to define a fluid flow which is integrated to obtain a mapping representing deformations in anatomy from the base amplitude. By varying the amplitudes according to the original breathing signal and applying these deformations at each measured amplitude, the trajectories of individual points in the anatomy are tracked.

#### B.2 Accounting for hysteresis motion with the 4D TDR ($4D\;TDR_{Hysteresis}$)

The phantom challenges any DIR or 4D TDR algorithm by its natural tissue hysteresis conditions. For a periodic breathing pattern under conditions of hysteresis, a given voxel's trajectory essentially corresponds to tracing one half of an approximately elliptical 3D path whenever the breathing signal is rising (exhale) and the other half when the breathing signal is falling (inhale). Langner and Keall^(^ ^16^ ^)^ investigated 5D image reconstruction using two‐parameter binning methods and found that the time derivative of the breathing signal, in combination with the breathing signal itself, gave the best parameterization. The time derivative of the RPM signal is obtained by applying a low‐pass filter to remove noise, then computing the time derivative using the central difference method. Therefore, the 4D ${\text{TDR}}_{\text{Hysteresis}}$ received partitioned data (i.e., two disjoint sets — those with positive breathing signal derivative and those with negative breathing signal derivative). The resulting two, amplitude‐index motion estimates were then jointly estimated, along with a common base image, estimating motion on each side of the hysteresis loop. This joint estimate was seen to produce an accurate motion path estimate and a more accurate base image.

#### B.3 Electronic masking of fiducials in porcine liver

To avoid the possibility of the deformation algorithm benefitting from the unfair advantage of being ‘driven’ by the implanted fiducials, all 4D CT image data were copied and fiducial locations were masked in the second dataset. Images containing fiducials or clearly visible artifacts of fiducials were processed prior to being submitted to the 4D TDR. In the original slice data, a rectangular region encompassing the streak artifacts centered around each fiducial was outlined, and a homogeneous region of equivalent size, containing unaliased liver tissue, was chosen in the same slice and tiled into the erased region. After tiling and successful visual verification of fiducial masking, pixels along the edges of the masked region were blended with their original values using a Tukey (cosine‐tapered) window function. This resulted in the slices being effectively masked of evidence of the fiducials while retaining the texture of homogenous liver tissue. Fiducials were erased in all slices ±12.5 mm along the superior and inferior direction.

## III. RESULTS

### A. Comparison of 4D CT measurement to EMT tracking measurement

**Figure 4:**
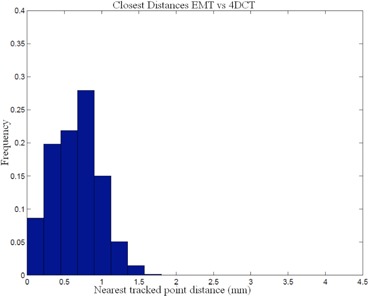
Comparison of EMT measurement of fiducial positions vs. 4D CT marker tracking for sinusoidal animation.

**Figure 5:**
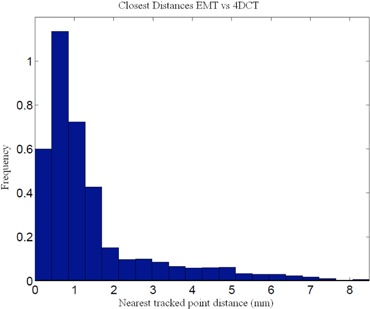
Comparison of EMT measurement of fiducial positions vs. 4DCT marker tracking for patient trace animation.

**Figure 6:**
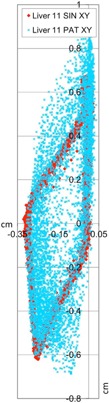
EMT point clouds for sin (red) and pat (blue) animation of the same porcine liver in two dimensions.

In order to ensure that the ‘ground truth’ 4D CT measurement accurately characterizes liver deformation without phase binning errors, we compared the measured 4D CT location of the EMT fiducials against the measured EMT traces. The accuracy of the EMT system has been reported to be less than 0.5 mm in any direction for motion speeds of up to 3 cm/s.^(^ ^17^ ^)^ Our comparison of the EMT and 4D CT motion tracks shows that 95% of the EMT points are within 1.2 mm of the 4D CT measurements (Table 2). A histogram of 3D distances of the measured EMT points to the closest interpolated 4D CT point of both 4D CT datasets using the sinusoidally (sin) animated liver indicates good agreement between the two measurement techniques, as depicted in Fig. 4, thereby confirming the accuracy of the 4D CT measurement and the reproducibility of motion. The same porcine liver, animated with a recorded patient (pat) trace captured with the RPM device, shows a greater variation of tissue motion due to the changes in each breath. Figure 5 shows a histogram of point distances between 4D CT and EMT for a patient trace porcine liver phantom animation. The difference in sinusoidal and patient trace animation can be seen in Fig. 6, which depicts an example of the EMT point clouds for sinusoidal (red) and patient (blue) animation of the porcine liver in two dimensions. While the EMT measurement points for the sinusoidal animation are clustered, clear, and distinct, the patient trace animated measurement (blue) shows a wider spread of motion in line with the animation variation.

**Table 2:** Percentile distances of the EMT trace points in millimeter distance to 4D CT points.

*Percentile of EMT Points*	*4D CT Data (measured)*
95%	1.2 mm
99%	1.4 mm

### B. Measurement errors

For the measurement of the coordinates of the two stationary markers in the phantom in all 20 binned CT sets, the fiducials' position in superior–inferior direction can vary based on slice thickness, which imposes an uncertainty of half the slice dimension (i.e., 0.675 mm). Our error analysis revealed that the standard deviation of fiducial position measurements is submillimeter. We were able to localize the center of a stationary BB from all CT phases with 0.1 mm standard deviation at 1.25 mm slice thickness with the phantom.

The maximum phase sampling error (MPSE) for the 4D CT datasets after binning, as stated by the AW software, was 1% indicating correct phase assignment.

**Figure 7:**
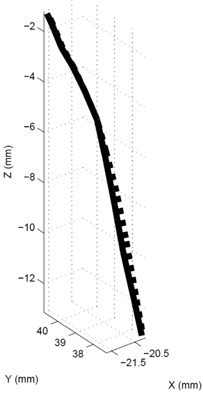
4D TDR tracks calculated from 4D CT (straight line) and 4D CT with masked fiducials (dotted lines).

Figure 7 displays the 4D TDR estimations of the 4D CT dataset with fiducials (lined track) and the respective calculation with fiducials masked (dotted track). For same datasets, negligible differences are found, as shown in Fig. 7, for one representative fiducial's track. The average deviation between the two corresponding 4D TDR motion paths was less than 0.1 mm, thus indicating that high‐contrast fiducials do not drive the 4D TDR and that masking the fiducials is acceptable. For the purpose of this article, we therefore continue with the masked dataset results only.

### C. 4D CT measurement and 4D TDR prediction without breathing signal derivative

**Figure 8:**
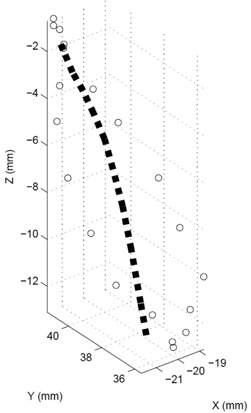
Tracks calculated with 4D TDR (dotted line) compared with 4D CT measurements (circles). Note the hysteresis shown in the 4D CT measurement.

Human tissue exhibits hysteresis during respiration.^(^ ^18^ ^,^ ^19^ ^)^ It is reasonable to anticipate challenges to a DIR or 4D TDR to correctly model such behavior. Based on the deviation vector between each of the measured fiducial positions (4D CT) and the corresponding 4D TDR predicted position for the two sinusoidal animated 4D CT scans, it becomes clear that the accuracy of prediction of the 4D TDR algorithm suffers, especially when passing the center portion of the motion hysteresis (see Fig. 8). The roughly elliptical track of a moving fiducial, represented by the open circles in Fig. 8, as measured from the 4D CT is indicative of the natural porcine liver tissue hysteresis. This particularly valuable feature of the porcine liver phantom utilized here allows for a precise characterization of a tissue hysteresis.

**Figure 9:**
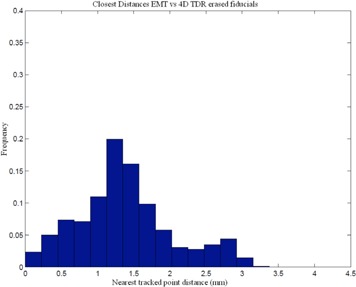
Comparison of EMT measurement of fiducial positions vs. 4D TDR predicted from the 4D CT dataset.

Table 3 presents comparisons of the 4D TDR results to the acquired sinusoidal animated 4D CT dataset. While the average error is low (1.9 mm) for the 3D distances between 4D TDR and 4D CT, it is noted that maximum deviations from a measured position could be as large as 4.5 mm for specific locations. Table 4 shows the 95% and 99% percentile of the EMT point‐cloud compared to 4D TDR predicted motion tracks with the calculation resulting in a 95% percentile of EMT points up to 2.8 mm. Figure 9 is a graphical representation of the 4D TDR to EMT match.

**Table 3:** 3D deviation of the 4D TDR and ${\text{TDR}}_{\text{Hysteresis}}$ vs. the 4D CT‐measured points.

*Sum 3D Vector* Δ *(mm)*		
*GE AW Phase*	$4D\;TDR_{Hysteresis}$	*4D TDR*
0%	0.87	1.22
5%	0.36	1.10
10%	0.61	0.54
15%	0.35	1.15
20%	0.59	1.64
25%	0.80	2.11
30%	0.71	1.95
35%	1.03	1.26
40%	0.70	1.14
45%	0.61	0.98
50%	1.12	0.93
55%	0.30	0.44
60%	0.15	0.48
65%	0.86	1.83
70%	0.70	2.46
75%	1.36	3.84
80%	1.90	4.31
85%	2.04	4.54
90%	1.75	3.22
95%	0.95	1.97
3D Vector	0.89	1.86
3D Vector Max Error (mm)	2.04	4.54

**Table 4:** Percentile distances of the EMT trace points in millimeter 3D distance to the 4D TDR.

*Percentile of EMT Points*	*3D Distance Between EMT and 4D TDR*
95%	2.75 mm
99%	2.97 mm

### D. Comparison of EMT tracking to the 4D ${\text{TDR}}_{\text{Hysteresis}}$ prediction (i.e., with breathing trace derivative)

**Figure 10:**
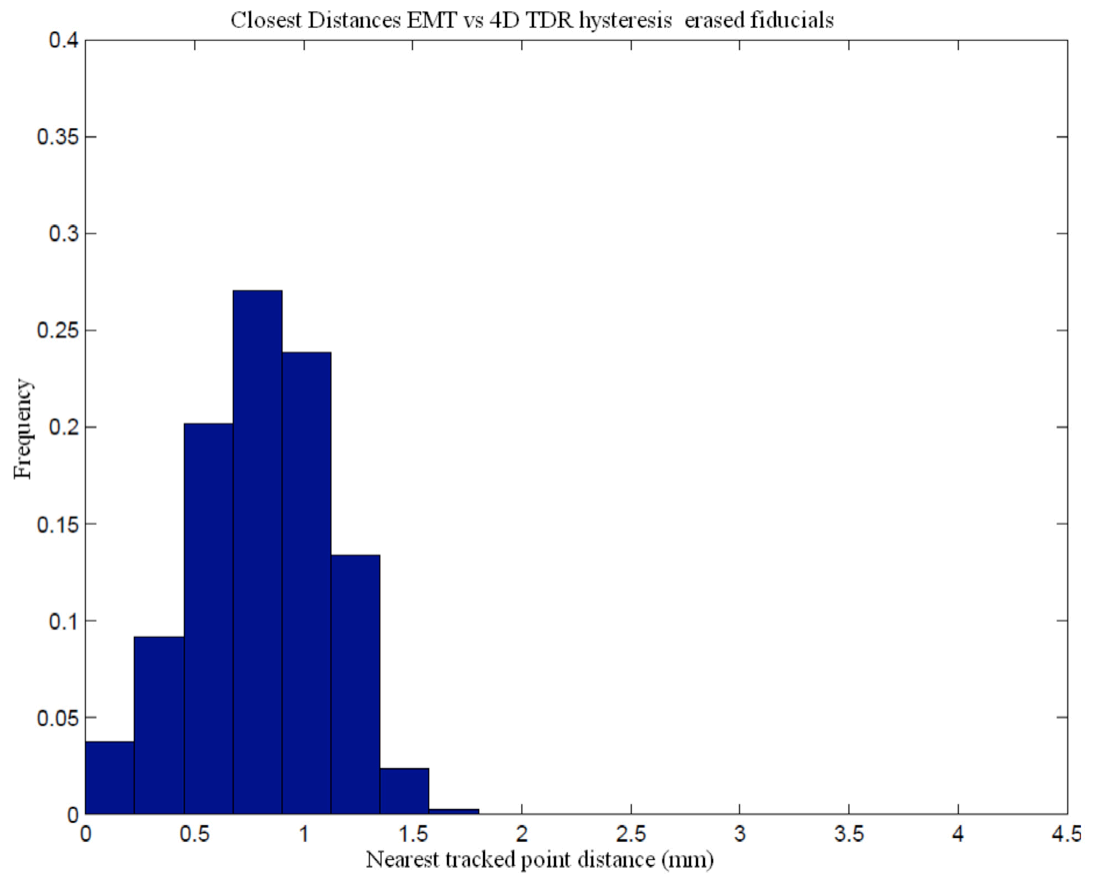
Comparison of 4D ${\text{TDR}}_{\text{Hysteresis}}$ predicted vs. EMT measurement of fiducial positions (mm).

The 4D ${\text{TDR}}_{\text{Hysteresis}}$ solution, incorporating the RPM time derivative, results in a 0.9 mm average 3D distance from the measured sinusoidal animated 4D CT‐phase datasets with fiducials. All 4D CT point distances for representative CT‐phase bins are presented in Table 3. We note that a direct consequence of using the 4D TDR means that there is no error‐free phase, since all data are compared to an average error. The distances for the 95% and 99% percentile of EMT points to the 4D ${\text{TDR}}_{\text{Hysteresis}}$ solution and to the measured 4D CT data are shown in Tables 3 and 5. A substantial reduction in the average and maximum error between measurement and prediction was experienced applying the 4D ${\text{TDR}}_{\text{Hysteresis}}$ compared to the 4D TDR. The 4D ${\text{TDR}}_{\text{Hysteresis}}$ results in Table 5 are close to the actual measurement comparison listed in Table 2. A histogram representation of the EMT point cloud matched to the 4D ${\text{TDR}}_{\text{Hysteresis}}$ estimate is displayed in Fig. 10, which compares favorably with the distances measured between EMT and 4D CT data shown in Fig. 4.

**Table 5:** Percentile of the EMT trace points in millimeter distance to the 4D ${\text{TDR}}_{\text{Hysteresis}}$ and to measured 4D CT data.

*Percentile of EMT Points*	$4D\;TDR_{Hysteresis}$	*4D CT Data (measured)*
95%	1.01 mm	1.18 mm
99%	1.15 mm	1.39 mm

### E. Comparison of EMT tracking to the 4D ${\text{TDR}}_{\text{Hysteresis}}$ prediction with patient trace‐based animation

**Figure 11:**
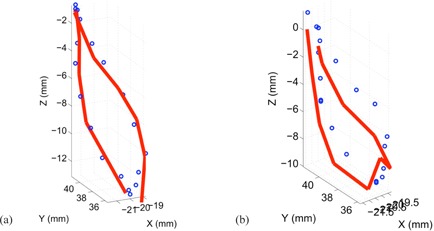
EMT point cloud distances to 4D CT measurement (circles) and 4D TDR (line) estimation for pat animated porcine liver: (a) the sin‐animated liver measurement; (b) the pat animated liver measurement.

The maximum phase sampling error (MPSE) for the patient‐trace animated 4D CT dataset after binning, as stated by the AW software, was 13%, indicating average phase assignment error as it is experienced in real patient data at any day. Based on the 4D ${\text{TDR}}_{\text{Hysteresis}}$ solution, we plotted the measured 4D CT data and overlaid the calculated motion estimate, arriving at a hysteresis motion. Figure 11 shows both motion estimates from the 4D ${\text{TDR}}_{\text{Hysteresis}}$ (sin and pat animation) for comparison, using the same liver. The circles represent the measured 4D CT fiducial locations.

**Figure 12:**
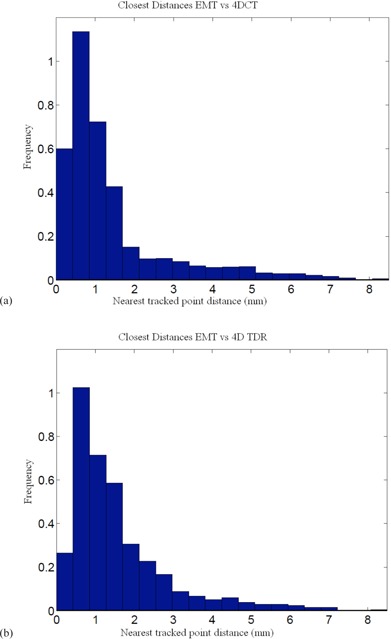
EMT point cloud distances to 4D CT measurement (a) and 4D ${\text{TDR}}_{\text{Hysteresis}}$ (b) estimation for pat animated porcine liver.

Numeric data, representing the 3D distance between measured 4D CT and 4D ${\text{TDR}}_{\text{Hysteresis}}$ is presented in Table 6. We note that a direct consequence of using the 4D ${\text{TDR}}_{\text{Hysteresis}}$ with patient trace animation is the slightly higher average error. Similarly, the distances for the 95% and 99% percentile of EMT points to the 4D ${\text{TDR}}_{\text{Hysteresis}}$ solution and to the measured 4D CT data, shown in Table 7, are substantially bigger due to the greater motion variation. The spread of EMT measurements (i.e., the EMT cloud pictured in Fig. 6) result directly from the variation induced. A histogram representation of the EMT point cloud matched to the 4D ${\text{TDR}}_{\text{Hysteresis}}$ estimate and the measured 4D CT of the patient trace animation is displayed in Figs. 12(a) and 12(b).

**Table 6:** 4D ${\text{TDR}}_{\text{Hysteresis}}$ vs. the patient trace 4D CT measured points.

*Sum 3D Vector* Δ *(mm)*	
*GE AW Phase*	*Distance (mm)*
0%	0.65
5%	0.66
10%	1.32
15%	1.17
20%	1.31
25%	1.28
30%	0.82
35%	0.57
40%	0.90
45%	1.52
50%	1.07
55%	1.06
60%	1.09
65%	1.32
70%	1.59
75%	0.85
80%	0.41
85%	0.30
90%	0.67
95%	0.68
3D Vector	0.96
3D Vector Max Error (mm)	1.59

**Table 7:** Percentile of the EMT trace points in millimeter distance to the patient trace 4D ${\text{TDR}}_{\text{Hysteresis}}$ and to measured 4D CT data.

*Percentile of EMT Points*	$4D\;TDR_{Hysteresis}$ *with Fiducials*	*4D CT Data (measured)*
95%	4.52	4.87 mm
99%	6.42	6.74 mm

## IV. DISCUSSION

The challenge of mapping all of the individual, phase‐specific calculated dose distributions onto a common reference frame for summation into an accumulated 4D dose distribution is to avoid CT binning artifacts and to maintain voxel trueness. Numerous DIRs have been validated with respect to their end‐to‐end accuracy, leaving one to guess how well the motion path itself has been described. Even if motion is calculated from phase to phase, phase binning errors can unduly influence the motion path estimation, and/or the calculation of motion vectors over less than optimal number of binned CT phases can influence motion estimates. For 4D cumulative dose volume histograms to be accurate enough to be deemed of clinical value, motion has to be validated at every point within the path, too.

DIR validation approaches have been limited to either mathematically‐based proofs and/or proof of performance by comparison with manually delineated organ contours derived from retrospective patient datasets. Highly variable breathing patterns in real human subjects makes acquisition of reliable data, as well as accurate measurements of deformation, challenging. The effects of irregular breathing invariably cause phase‐binning algorithms to mislabel phase to images,^(^ ^20^ ^–^ ^22^ ^)^ thus causing nontrivial spatial and temporal artifacts and resulting errors in DIRs. To avoid the limitations of patient‐based studies on accuracy within the motion path and binning artifacts, the biologically realistic phantom‐based validation is performed here using highly reproducible liver tissue motion. The benefits of operating in a completely realistic tissue environment, capable of repeatedly modeling and characterizing real tissue hysteresis and deformation, improve validation efforts. The highly reproducible nature of the phantom's liver tissue motion reduces vulnerability to the inaccurate representation of motion in 4D CT which occurs in irregularly breathing patients. By avoiding simplified tissue modeling approaches, we strived to counter challenges to the evaluation through imaging limitations on real patients. The 4D TDR algorithm's bin‐less approach, therefore, is decoupled from the binned CT measured data.

Interestingly, more recent reports have described the use of phantoms to address the limitations of patient studies and, thus, improve on the data acquisition needed for accurate validation of deformation algorithms. The materials used in these studies were either rigid,^(^ ^23^ ^)^ or sponge‐like,^(^ ^23^ ^,^ ^24^ ^)^ or a mixture of both,^(^ ^24^ ^,^ ^25^ ^)^ with translational motion being the predominant motion modeled. Unfortunately, the use of nontissue materials in these experiments does not allow for fully accurate modeling of the realistic tissue‐deformable voxels, nor do they yield entirely realistic motion, such as hysteresis. Previous phantoms have therefore been limited in the extent to which they could accurately validate deformation estimates due to their somewhat simplified tissue modeling approaches. Using the porcine liver with embedded fiducials, we accurately characterize tissue hysteresis using 4D CT. The characterized hysteresis pattern allows for study of tissue behaviors when faced with irregular motion. The fit of the 4D ${\text{TDR}}_{\text{Hysteresis}}$ solution to the full complex motion path to measured 4D CT and EMT data to within less than 2 mm on average can therefore be validated. The average error presented here applies to any point within the motion path and not only to motion/deformation endpoints.

Multiple studies^(^ ^22^ ^,^ ^26^ ^,^ ^27^ ^)^ have used limited sets of retrospective patient data (between 2 and 5) from 4D CT acquisition to validate their respective deformation models. These and other authors have gleaned their results from centroid‐to‐centroid comparison,(5) volume‐overlap‐comparison, image cross‐correlation, distance to agreement of manually/visually identified landmarks or organ‐contours in 2D, as well as in 3D, isointensity contours,^(^ ^7^ ^,^ ^28^ ^)^ and other similar measures. Average end‐to‐end errors in such studies are reported to range from 2–3 mm, but it is important to note that none of the aforementioned methods report on the complete motion path accuracy. While hysteresis has been observed and reported in patients,^(^ ^18^ ^,^ ^19^ ^)^ reproducing and accurately imaging the same hysteresis has thus far has been very challenging in patients. We note that, while our mean error over all phases was below 2 mm, we measured errors as large as 7.0 mm for individual CT phases. These larger deviations coincided with positions marking the widest cross section of the phantom‐produced hysteresis, highlighting the need for complete motion‐path (i.e., including hysteresis) characterization in patients. The methods used for these studies are in contrast to the biologically realistic, phantom‐based validation performed here which is less vulnerable to the inaccurate representation of motion due to irregularly breathing patients

As expected, the greatest opportunities for error reduction in deformation estimation were found at the widest part of the tissue hysteresis loop where the error was initially the greatest. The original 4D TDR prediction experienced the greatest error of prediction in the 25%–30% and 75%–80% phase range, while the improved 4D ${\text{TDR}}_{\text{Hysteresis}}$ experienced significantly less error of prediction at these phase ranges, reducing by almost one half the error between measurement and calculation (~0.5−1.3 mm error).

**Figure 13:**
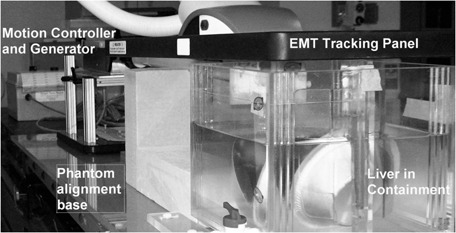
Real‐tissue phantom placed under the EMT wand for motion tracking.

We found the realistic tissue deformation phantom to be a valuable tool to prove or to improve on the accuracy of the 4D TDR. Our efforts to acquire a complete, nonphase‐sampled, 4D TDR dataset from another institution to confirm above results have thus far been unsuccessful. Current shared datasets available in the public domain are phase‐sampled and, thus, would negate the most important advantage the 4D TDR offers over other algorithms. Additional verification of motion public domain data using the phantom in conjunction with an EMT tracking system as shown in Fig. 13 would not be possible either.

## V. CONCLUSIONS

Accurate validation over the whole motion path is essential in evaluating the usefulness of any DIR or 4D TDR intended for use in 4D dose calculation methods. The phantom allowed the quantification of a specific hysteresis motion path using the 4D TDR under realistic conditions of regular and irregular animation. While endpoint accuracy was comparable to other publications, overall accuracy of the whole sinusoidal motion path of the 4D ${\text{TDR}}_{\text{Hysteresis}}$ was measured to have a 1.4 mm average error compared to the measured data of 4D CT. In comparison to the EMT data point cloud, we observed 95% of points to be within 1 mm of the 4D ${\text{TDR}}_{\text{Hysteresis}}$ solution. The 4D ${\text{TDR}}_{\text{Hysteresis}}$ traces matched 95% of the EMT trace within 1.6 mm when using the sinusoidal signal and 4.5 mm when using the patient trace for animation.

The biologically realistic porcine liver phantom accurately represents and allows characterization of a tissue hysteresis. The fit of the 4D TDR estimated motion trace to the motion path as measured by 4D CT and EMT can therefore be validated accurately for a sinusoidal and patient trace animated porcine liver. The average error presented here applies to any point within the motion path, not only to motion/deformation endpoints.

## Supporting information

Supplementary Material

## References

[1] Zeng R . Estimating respiratory motion from CT images via deformable models and priors [dissertation]. Department of Electrical Engineering: Systems. Ann Arbor, MI: University of Michigan; 2007.

[2] Rueckert D , Aljabar P , Heckemann RA , Hajnal JV , Hammers A . Diffeomorphic registration using B‐splines. In: Medical Image Computing and Computer‐Assisted Intervention – MICCAI 2006. Lecture Notes in Computer Science. 2006;4191:702–09. 10.1007/11866763_86 17354834

[3] Rohlfing T , Maurer CR Jr , Bluemke DA , Jacobs MA . Volume‐preserving nonrigid registration of MR breast images using free‐form deformation with an incompressibility constraint. IEEE Trans Med Imaging, 2003;22(6):730–41. 12872948 10.1109/TMI.2003.814791

[4] Foskey M , Davis B , Goyal L , et al. Large deformation three‐dimensional image registration in image‐guided radiation therapy. Phys Med Biol. 2005;50(24):5869–92. 16333161 10.1088/0031-9155/50/24/008

[5] Pevsner A , Davis B , Joshi S , et al. Evaluation of an automated deformable image matching method for quantifying lung motion in respiration‐correlated CT images. Med Phys. 2006;33(2):369–76. 16532942 10.1118/1.2161408

[6] Hinkle J , Fletcher PT , Wang B , Salter B , Joshi S . 4D MAP image reconstruction incorporating organ motion, p. 676–87. In: IPMI 2009: Proceedings of the 21^st^ International Conference on Information Processing in Medical Imaging. New York: Springer; 2009. 10.1007/978-3-642-02498-6_56 19694303

[7] Rohlfing T , Maurer CR Jr , O'Dell WG , Zhong J . Modeling liver motion and deformation during the respiratory cycle using intensity‐based nonrigid registration of gated MR images. Med Phys. 2004;31(3):427–32. 15070239 10.1118/1.1644513

[8] Zhang Q , Pevsner A , Hertanto A , et al. A patient‐specific respiratory model of anatomical motion for radiation treatment planning. Med Phys. 2007;34(12):4772–81. 18196805 10.1118/1.2804576

[9] Brock KK . Results of a multi‐institution deformable registration accuracy study (MIDRAS). Int J Radiat Oncol Biol Phys. 2010;76(2):583–96. 19910137 10.1016/j.ijrobp.2009.06.031

[10] Szegedi M , Rassiah‐Szegedi P , Fullerton G , Wang B , Salter B . A proto‐type design of a real tissue phantom for the validation of deformation algorithm and 4D dose calculations. Phys Med Biol. 2010;55(13):3685–99. 20530851 10.1088/0031-9155/55/13/008

[11] Szegedi M , Fullerton G , Rassiah‐Szegedi P , Salter B . Characterization of liver motion based on implanted markers [abstract]. Med Phys. 2009;36(6):2506.

[12] Vedam SS , Kini VR , Keall PJ , Ramakrishnan V , Mostafavi H , Mohan R . Quantifying the predictability of diaphragm motion during respiration with a noninvasive external marker. Med Phys. 2003;30(4):505–13. 12722802 10.1118/1.1558675

[13] Kubo HD and Hill BC . Respiration gated radiotherapy treatment: a technical study. Phys Med Biol. 1996;41(1):83–92. 8685260 10.1088/0031-9155/41/1/007

[14] Kubo HD , Len PM , Minohara S , Mostafavi H . Breathing‐synchronized radiotherapy program at the University of California Davis Cancer Center. Med Phys. 2000;27(2):346–53. 10718138 10.1118/1.598837

[15] Pan T , Lee TY , Rietzel E , Chen GT . 4D‐CT imaging of a volume influenced by respiratory motion on multi‐slice CT. Med Phys. 2004;31(2):333–40. 15000619 10.1118/1.1639993

[16] Langner U and Keall P . Accuracy in the localization of thoracic and abdominal tumors using respiratory displacement, velocity, and phase. Med Phys. 2009;36(2):386–93. 19291977 10.1118/1.3049595 PMC2736730

[17] Balter JM , Wright JN , Newell LJ , et al. Accuracy of a wireless localization system for radiotherapy. Int J Radiat Oncol Biol Phys. 2005;61(3):933–37. 15708277 10.1016/j.ijrobp.2004.11.009

[18] Remmert G , Biederer J , Lohberger F , Fabel M , Hartmann GH . Four‐dimensional magnetic resonance imaging for the determination of tumour movement and its evaluation using a dynamic porcine lung phantom. Phys Med Biol. 2007;52(18):N401–N415. 17804874 10.1088/0031-9155/52/18/N02

[19] Boldea V , Sharp GC , Jiang SB , Sarrut D . 4D‐CT lung motion estimation with deformable registration: quantification of motion nonlinearity and hysteresis. Med Phys. 2008;35(3):1008–18. 18404936 10.1118/1.2839103

[20] Mutaf YD , Antolak JA , Brinkmann DH . The impact of temporal inaccuracies on 4DCT image quality. Med Phys. 2007;34(5):1615–22. 17555243 10.1118/1.2717404

[21] Pan T . Comparison of helical and cine acquisitions for 4D‐CT imaging with multislice CT. Med Phys. 2005;32(2):627–34. 15789609 10.1118/1.1855013

[22] Rietzel E , Chen GTY . Improving retrospective sorting of 4D computed tomography data. Med Phys. 2006;33(2):377–79. 16532943 10.1118/1.2150780

[23] Kashani R , Hub M , Kessler ML , Balter JM . Technical note: a physical phantom for assessment of accuracy of deformable alignment algorithms. Med Phys. 2007;34(7):2785–88. 17821985 10.1118/1.2739812

[24] Vinogradskiy Y , Balter P , Followill DS , Alvarez PE , White RA , Starkschall G . Verification of four‐dimensional photon dose calculations. Med Phys. 2009;36(8):3438–47. 19746777 10.1118/1.3157233

[25] Serban M , Heath E , Stroian G , Collins DL , Seuntjens J . A deformable phantom for 4D radiotherapy verification: design and image registration evaluation. Med Phys. 2008;35(3):1094–102. 18404944 10.1118/1.2836417

[26] Flampouri S , Jiang SB , Sharp GC , Wolfgang J , Patel AA , Choi NC . Estimation of the delivered patient dose in lung IMRT treatment based on deformable registration of 4D‐CT data and Monte Carlo simulations. Phys Med Biol. 2006;51(11):2763–79. 16723765 10.1088/0031-9155/51/11/006

[27] Heath E , Collins DL , Keall PJ , Dong L , Seuntjens J . Quantification of accuracy of the automated nonlinear image matching and anatomical labeling (ANIMAL) nonlinear registration algorithm for 4D CT images of lung. Med Phys. 2007;34(11):4409–21. 18072506 10.1118/1.2795824

[28] Rohlfing T , Maurer CR Jr , O'Dell WG , Zhong J . Modeling liver motion and deformation during the respiratory cycle using intensity‐based free‐form registration of gated MR images. Proc SPIE. 2001;4319:337–48. 10.1118/1.1644513 15070239

