# Supplementary figures and images for: Four‐dimensional tissue deformation reconstruction (4D TDR) validation using a real tissue phantom
Source: J Appl Clin Med Phys. 2013 Jan 7;14(1):115–32. doi: 10.1120/jacmp.v14i1.4012 (PMC5713919; doi:10.1120/jacmp.v14i1.4012)

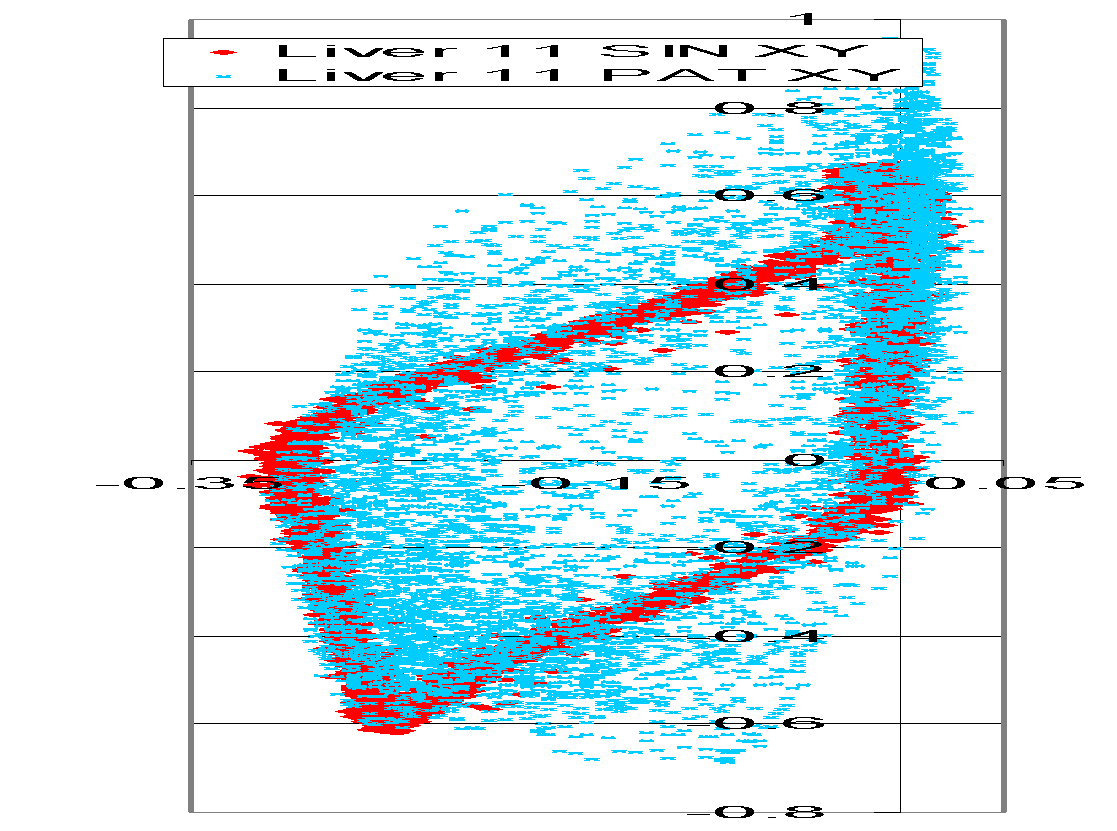

Supplement: Supplementary file 1 — Supplementary Material [file ACM2-14-115-s001.tif]
